# The genome sequence of the black-footed limpet, *Patella depressa *(Pennant, 1777)

**DOI:** 10.12688/wellcomeopenres.20687.1

**Published:** 2024-02-19

**Authors:** Stephen J. Hawkins, Nova Mieszkowska, Rob Mrowicki, Marine Biological Association Genome Acquisition Lab, Darwin Tree of Life Barcoding collective, Wellcome Sanger Institute Tree of Life Management, Samples and Laboratory team, Wellcome Sanger Institute Scientific Operations: Sequencing Operations, Wellcome Sanger Institute Tree of Life Core Informatics team, Tree of Life Core Informatics collective, Darwin Tree of Life Consortium

**Affiliations:** 1 The Marine Biological Association, Plymouth, England, UK

**Keywords:** Patella depressa, black-footed limpet, genome sequence, chromosomal, Patellogastropoda

## Abstract

We present a genome assembly from an individual *Patella depressa* (the black-footed limpet; Mollusca; Gastropoda; Patellogastropoda; Patellidae). The genome sequence is 683.7 megabases in span. Most of the assembly is scaffolded into 9 chromosomal pseudomolecules. Gene annotation of this assembly on Ensembl identified 20,502 protein coding genes.

## Species taxonomy

Eukaryota; Metazoa; Eumetazoa; Bilateria; Protostomia; Spiralia; Lophotrochozoa; Mollusca; Gastropoda; Patellogastropoda; Patelloidea; Patellidae; *Patella*; *Patella depressa* (Pennant, 1777) (NCBI:txid87960).

## Background

*Patella depressa* (Pennant, 1777) (in the past called *P. intermedia* Murray in Knapp, 1857 – junior synonym) has a confused taxonomy, although *P. depressa* has become the widely accepted name ( https://www.marinespecies.org/aphia.php?p=taxdetails&id=151374). *P. depressa* occurs from Senegal in north Africa and has been found on Anglesey in north Wales, but the current leading range edge is Porth Oer in north Wales. It is absent from Ireland as it did not cross the Irish Sea at the end of the last Ice Age. In the English Channel breeding populations occur as far east as the Isle of Wight and the Cotenin Peninsula. It occurs primarily on moderately exposed and exposed shores, rarely being found in seaweed-dominated sheltered shores in northern France and the British Isles. It is the dominant mid- and high-shore limpet from north Africa to southern Brittany, giving way to *Patella ulysipponensis* lower down the shore and in rockpools. In response to climate warming, it has become the dominant limpet in the mid and upper reaches of shores in southwest England, and isolated individuals have been found east of the Isle of Wight. The northern leading range edge in North Wales retracted from Anglesey south to the Lleyn Peninsula during the warm spell of the 1960s to mid 1980s ( Kendall *et al.*, 2004) and recolonisation beyond the Llyen has not occurred to date. It is absent from the Mediterranean and gives way to its sister species *Patella caerulea* to the east of the Alboran Front.

*Patella depressa* is capable of being a multiple brooder ( Ribeiro *et al.*, 2009). This pattern contrasts to only developing one brood in the cooler 1940s ( Orton & Southward, 1961). In response to recent warming *P. depressa* now has multiple broods in southwest England ( Moore *et al.*, 2011) with an extended breeding season from March to October. Further south in Portugal it is reproductively active for much of the year, with a short resting season in the summer ( Ribeiro *et al.*, 2009). *P. depressa* does not exhibit protandry, having equal proportions of males and females ( Borges *et al.*, 2015; Orton & Southward, 1961). *P. depressa* has a shorter larval life ( Ribiero, 2009) than *P. vulgata*, and can be reared without feeding, suggesting at least partial lecitrophy. This may restrict potential dispersal, explaining the lack of colonization of Ireland and allopatric speciation from *Patella caerulea* due to separation of Atlantic and Mediterranean populations.

*P. depressa* larvae settle in crustose coralline algae covered shallow rockpools, emerging from these nursery grounds after 1 to 3 years ( Bowman, 1981; Seabra *et al.*, 2020). Adults home, but unlike *P. vulgata* do not aggregate under fucoid clumps ( Moore *et al.*, 2007a). *P. depressa* grazing may be less efficient in controlling fucoid germlings than *P. vulgata* ( Moore *et al.*, 2007b). Exclusion experiments throughout Europe show *P. depressa* prevents algal colonisation in northern Spain plus central and southern Portugal ( Coleman *et al.*, 2006) where it is usually the only species of limpet present ( Boaventura *et al.*, 2002b). Intense intraspecific competition has been shown in *P. depressa* in Portugal ( Boaventura *et al.*, 2002a), especially between large and small size classes ( Boaventura *et al.*, 2003).

## Genome sequence report

**Figure 1.**
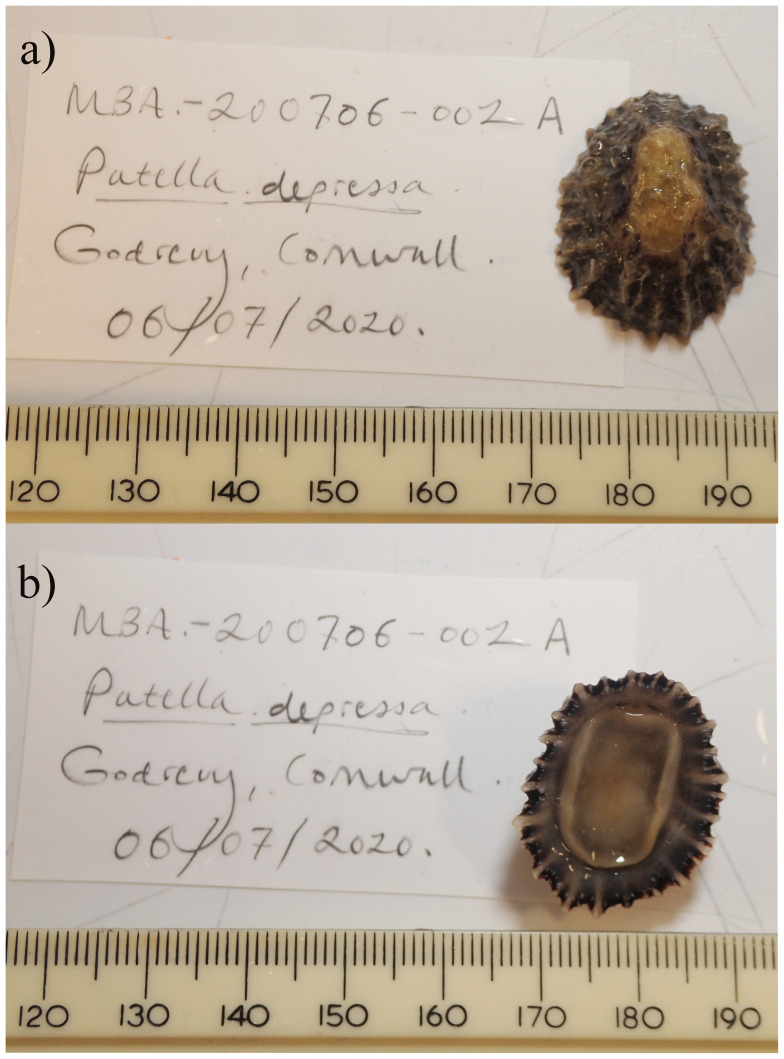
Photographs of the *Patella depressa* (xgPatDepr1) specimen used for genome sequencing.

The genome was sequenced from one *Patella depressa* ( Figure 1) collected from Godrevy, Cornwall, UK (50.24, –5.40). A total of 37-fold coverage in Pacific Biosciences single-molecule HiFi long reads and 93-fold coverage in 10X Genomics read clouds were generated. Primary assembly contigs were scaffolded with chromosome conformation Hi-C data. Manual assembly curation corrected 101 missing joins or mis-joins and removed 2 haplotypic duplications, reducing the scaffold number by 43.55%, and increasing the scaffold N50 by 52.61%.

**Figure 2.**
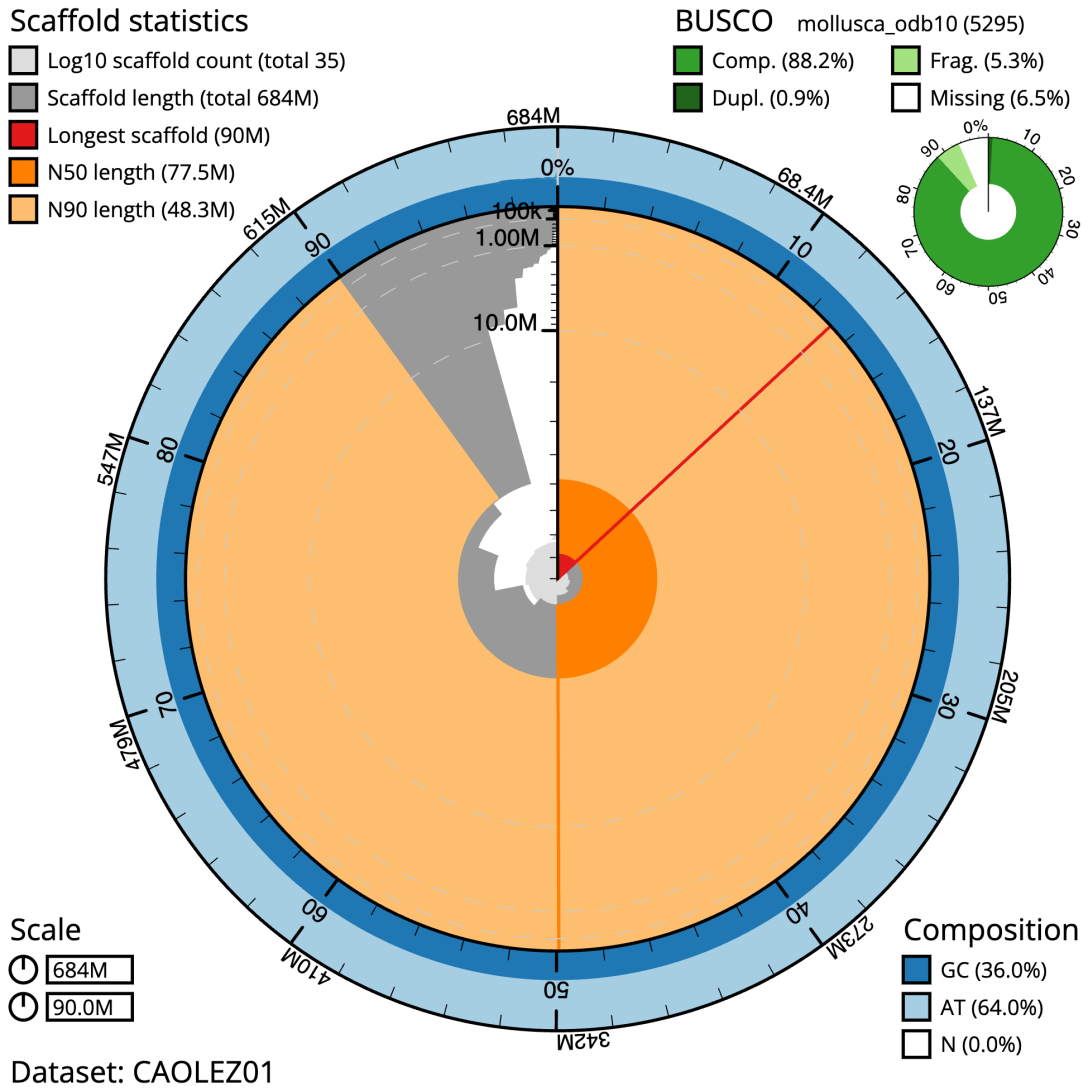
Genome assembly of *Patella depressa*, xgPatDepr1.1: metrics. The BlobToolKit Snailplot shows N50 metrics and BUSCO gene completeness. The main plot is divided into 1,000 size-ordered bins around the circumference with each bin representing 0.1% of the 683,711,263 bp assembly. The distribution of scaffold lengths is shown in dark grey with the plot radius scaled to the longest scaffold present in the assembly (90,014,739 bp, shown in red). Orange and pale-orange arcs show the N50 and N90 scaffold lengths (77,532,951 and 48,282,134 bp), respectively. The pale grey spiral shows the cumulative scaffold count on a log scale with white scale lines showing successive orders of magnitude. The blue and pale-blue area around the outside of the plot shows the distribution of GC, AT and N percentages in the same bins as the inner plot. A summary of complete, fragmented, duplicated and missing BUSCO genes in the mollusca_odb10 set is shown in the top right. An interactive version of this figure is available at https://blobtoolkit.genomehubs.org/view/Patella%20depressa/dataset/CAOLEZ01/snail.

**Figure 3.**
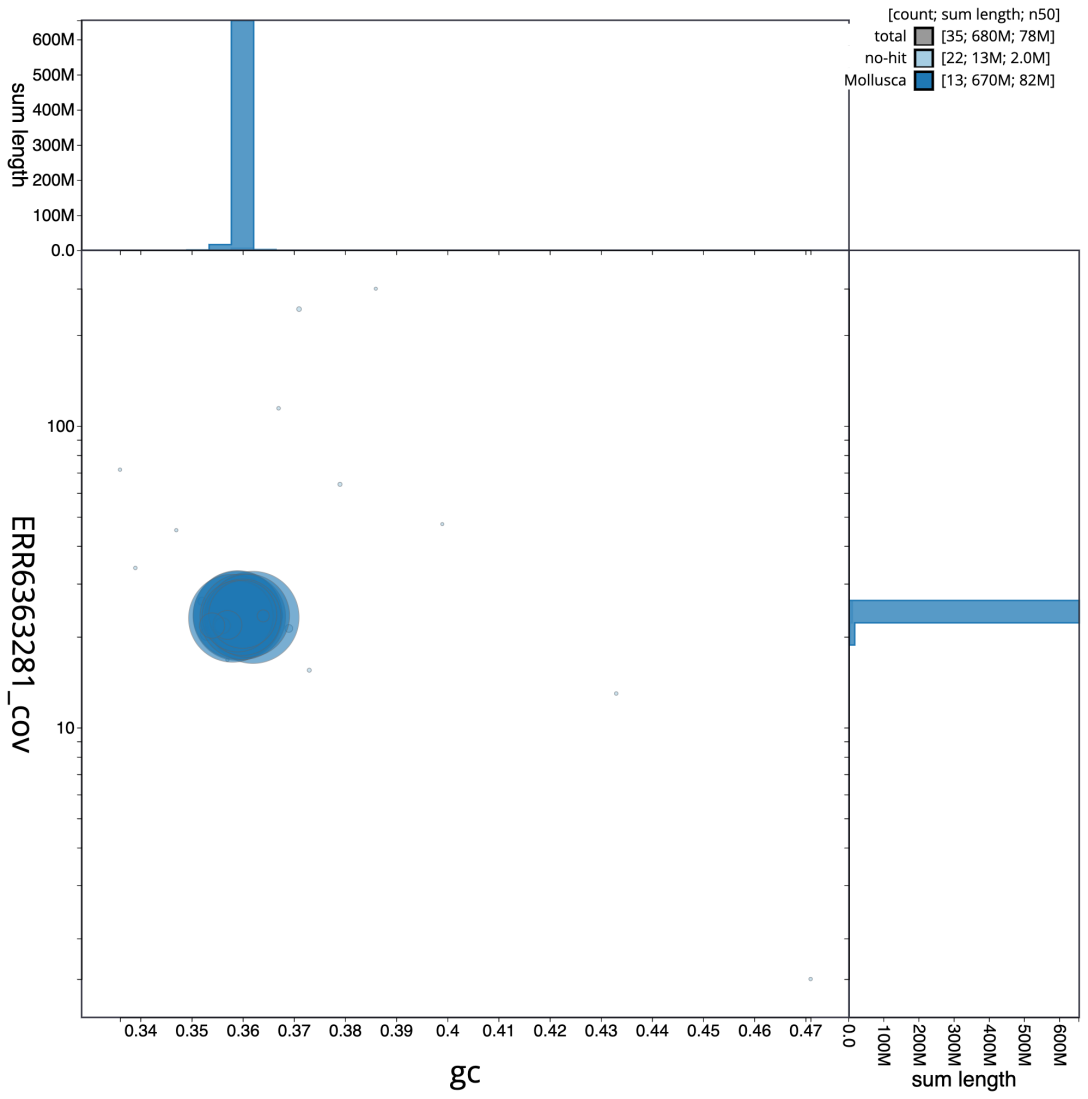
Genome assembly of *Patella depressa*, xgPatDepr1.1: BlobToolKit GC-coverage plot. Scaffolds are coloured by phylum. Circles are sized in proportion to scaffold length. Histograms show the distribution of scaffold length sum along each axis. An interactive version of this figure is available at https://blobtoolkit.genomehubs.org/view/Patella%20depressa/dataset/CAOLEZ01/blob.

**Figure 4.**
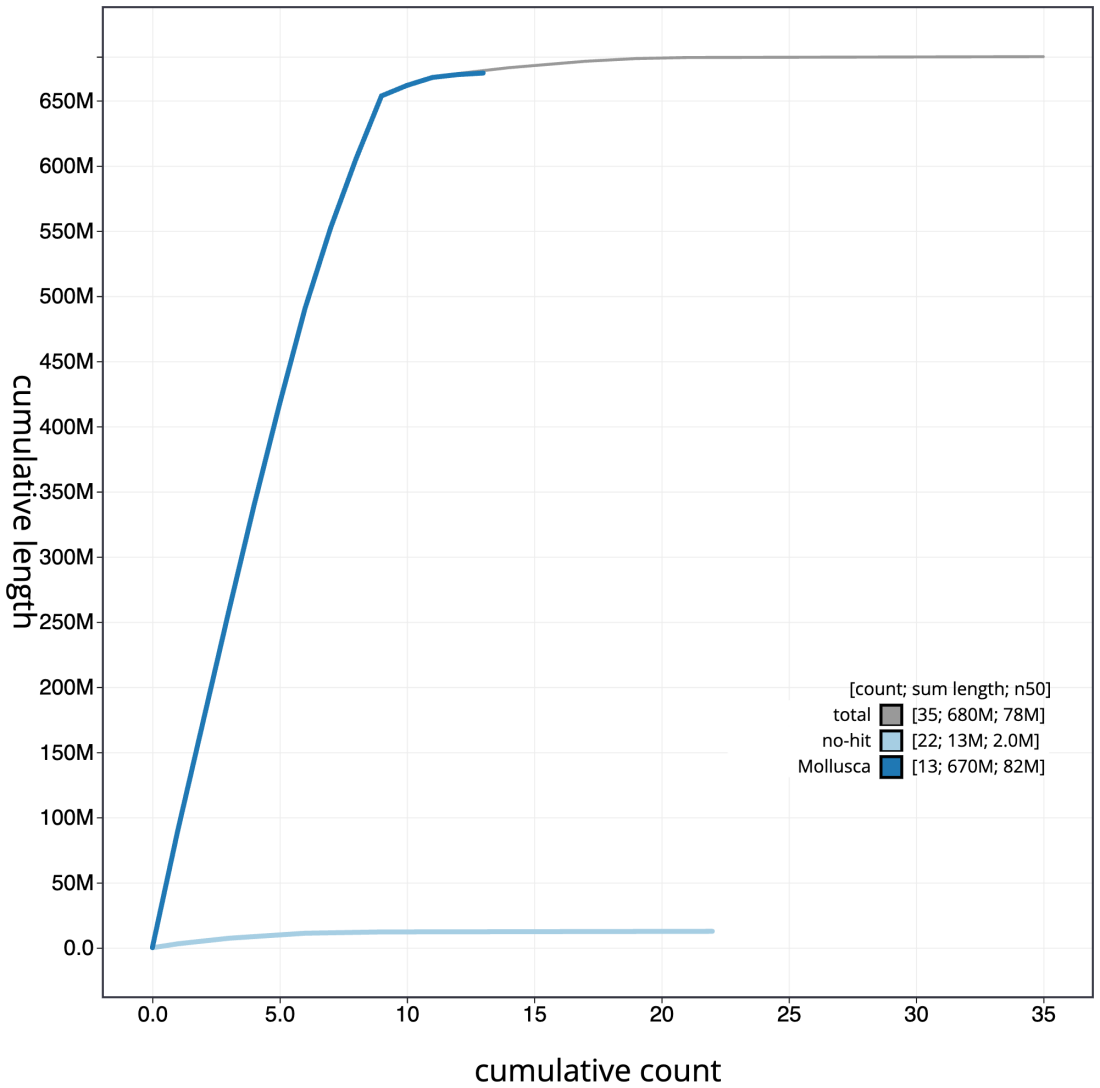
Genome assembly of *Patella depressa*, xgPatDepr1.1: BlobToolKit cumulative sequence plot. The grey line shows cumulative length for all scaffolds. Coloured lines show cumulative lengths of scaffolds assigned to each phylum using the buscogenes taxrule. An interactive version of this figure is available at https://blobtoolkit.genomehubs.org/view/Patella%20depressa/dataset/CAOLEZ01/cumulative.

**Figure 5.**
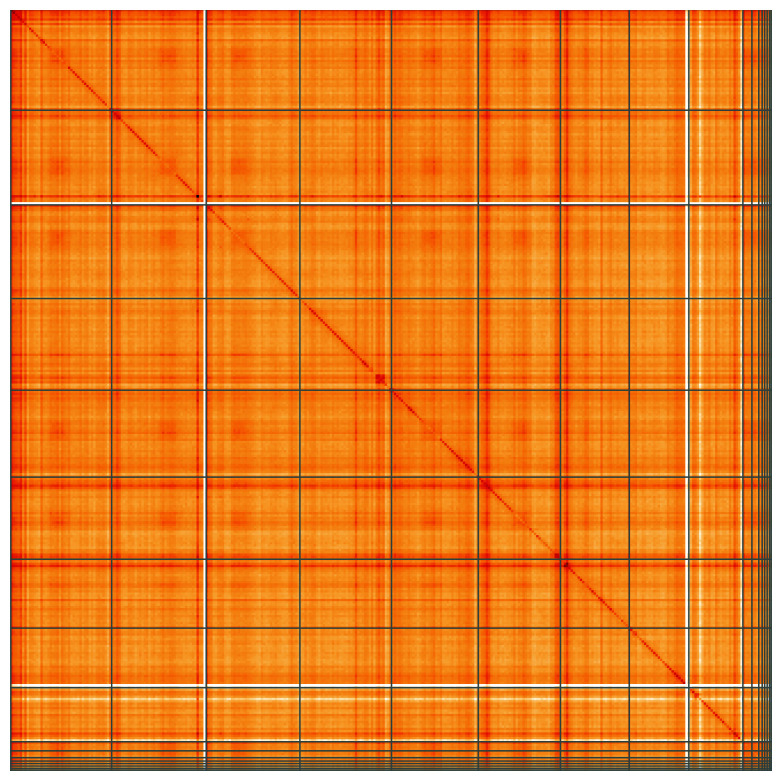
Genome assembly of *Patella depressa*, xgPatDepr1.1: Hi-C contact map of the xgPatDepr1.1 assembly, visualised using HiGlass. Chromosomes are shown in order of size from left to right and top to bottom. An interactive version of this figure may be viewed at https://genome-note-higlass.tol.sanger.ac.uk/l/?d=R0pa8NFgRziHwpvHMaztxg.

The final assembly has a total length of 683.7 Mb in 35 sequence scaffolds with a scaffold N50 of 77.5 Mb ( Table 1). The snailplot in Figure 2 provides a summary of the assembly statistics, while the distribution of assembly scaffolds on GC proportion and coverage is shown in Figure 3. The cumulative assembly plot in Figure 4 shows curves for subsets of scaffolds assigned to different phyla. Most (95.59%) of the assembly sequence was assigned to 9 chromosomal-level scaffolds. Chromosome-scale scaffolds confirmed by the Hi-C data are named in order of size ( Figure 5; Table 2). While not fully phased, the assembly deposited is of one haplotype. Contigs corresponding to the second haplotype have also been deposited. The mitochondrial genome was also assembled and can be found as a contig within the multifasta file of the genome submission.

**Table 1.** Genome data for *Patella depressa*, xgPatDepr1.1.

Project accession data		
Assembly identifier	xgPatDepr1.1	
Species	*Patella depressa*	
Specimen	xgPatDepr1	
NCBI taxonomy ID	87960	
BioProject	PRJEB45666	
BioSample ID	SAMEA7536239	
Isolate information	xgPatDepr1	
Assembly metrics *		*Benchmark*
Consensus quality (QV)	56.7	*≥ 50*
*k*-mer completeness	99.99%	*≥ 95%*
BUSCO **	C:88.2%[S:87.3%,D:0.9%], F:5.3%,M:6.5%,n:5,295	*C ≥ 95%*
Percentage of assembly mapped to chromosomes	95.59%	*≥ 95%*
Sex chromosomes	-	*localised homologous pairs*
Organelles	-	*complete single alleles*
Raw data accessions		
PacificBiosciences SEQUEL II	ERR6939215	
10X Genomics Illumina	ERR6363283, ERR6363280, ERR6363282, ERR6363281	
Hi-C Illumina	ERR6363279	
PolyA RNA-Seq Illumina	ERR9434988	
Genome assembly		
Assembly accession	GCA_948474765.1	
*Accession of alternate haplotype*	GCA_948474705.1	
Span (Mb)	683.7	
Number of contigs	117	
Contig N50 length (Mb)	13.9	
Number of scaffolds	35	
Scaffold N50 length (Mb)	77.5	
Longest scaffold (Mb)	90.0	
Genome annotation		
Number of protein-coding genes	20,502	
Number of non-coding genes	19,552	
Number of gene transcripts	57,683	

* Assembly metric benchmarks are adapted from column VGP-2020 of “Table 1: Proposed standards and metrics for defining genome assembly quality” from ( Rhie *et al.*, 2021). ** BUSCO scores based on the mollusca_odb10 BUSCO set using v5.3.2. C = complete [S = single copy, D = duplicated], F = fragmented, M = missing, n = number of orthologues in comparison. A full set of BUSCO scores is available at https://blobtoolkit.genomehubs.org/view/Patella%20depressa/dataset/CAOLEZ01/busco.

**Table 2.** Chromosomal pseudomolecules in the genome assembly of *Patella depressa*, xgPatDepr1.

INSDC accession	Chromosome	Length (Mb)	GC%
OX419715.1	1	90.01	36
OX419716.1	2	84.41	36
OX419717.1	3	83.61	36
OX419718.1	4	81.79	36
OX419719.1	5	77.53	36
OX419720.1	6	73.31	36
OX419721.1	7	61.48	36
OX419722.1	8	53.14	36
OX419723.1	9	48.28	36

The estimated Quality Value (QV) of the final assembly is 56.7 with *k*-mer completeness of 99.99%, and the assembly has a BUSCO v5.3.2 completeness of 88.2% (single = 87.3%, duplicated = 0.9%), using the mollusca_odb10 reference set ( *n* = 5,295).

Metadata for specimens, barcode results, spectra estimates, sequencing runs, contaminants and pre-curation assembly statistics are given at https://links.tol.sanger.ac.uk/species/87960.

## Genome annotation report

The *Patella depressa* genome assembly (GCA_948474765.1) was annotated using the Ensembl rapid annotation pipeline ( Table 1; https://rapid.ensembl.org/Patella_depressa_GCA_948474765.1/Info/Index). The resulting annotation includes 57,683 transcribed mRNAs from 20,502 protein-coding and 19,552 non-coding genes.

## Methods

### Sample acquisition and nucleic acid extraction

A *Patella depressa* (specimen ID MBA-200706-002A, ToLID xgPatDepr1) was collected by hand from Godrevy, Cornwall, UK (latitude 50.24, longitude –5.40) on 2020-07-06. The specimen was collected by Nova Mieszkowska and Rob Mrowicki (Marine Biological Association) and identified by Nova Mieszkowska, and then preserved in liquid nitrogen.

The workflow for high molecular weight (HMW) DNA extraction at the Wellcome Sanger Institute (WSI) includes a sequence of core procedures: sample preparation; sample homogenisation; DNA extraction; HMW DNA fragmentation; and fragmented DNA clean-up. The xgPatDepr1 sample was weighed and dissected on dry ice with tissue set aside for Hi-C sequencing ( Jay *et al.*, 2023). For sample homogenisation, muscle tissue was cryogenically disrupted using the Sample Homogenisation: Covaris cryoPREP ^®^ Automated Dry Pulverizer protocol ( Narváez-Gómez *et al.*, 2023). HMW DNA was extracted by means of the Manual MagAttract protocol ( Strickland *et al.*, 2023b). The DNA was sheared into an average fragment size of 12–20 kb in a Megaruptor 3 system with speed setting 30, following the HMW DNA Fragmentation: Diagenode Megaruptor ^®^3 for PacBio HiFi protocol ( Todorovic *et al.*, 2023). Sheared DNA was purified using solid-phase reversible immobilisation (SPRI) ( Strickland *et al.*, 2023a). In brief, the method employs a 1.8X ratio of AMPure PB beads to sample to eliminate shorter fragments and concentrate the DNA. The concentration of the sheared and purified DNA was assessed using a Nanodrop spectrophotometer and Qubit Fluorometer and Qubit dsDNA High Sensitivity Assay kit. Fragment size distribution was evaluated by running the sample on the FemtoPulse system.

RNA was extracted from xgPatDepr1 in the Tree of Life Laboratory at the WSI using the RNA Extraction: Automated MagMax™ *mir*Vana protocol ( do Amaral *et al.*, 2023). The RNA concentration was assessed using a Nanodrop spectrophotometer and Qubit Fluorometer using the Qubit RNA Broad-Range (BR) Assay kit. Analysis of the integrity of the RNA was done using the Agilent RNA 6000 Pico Kit and Eukaryotic Total RNA assay.

Protocols developed by the WSI Tree of Life laboratory are publicly available on protocols.io ( Denton *et al.*, 2023).

### Sequencing

Pacific Biosciences HiFi circular consensus and 10X Genomics read cloud DNA sequencing libraries were constructed according to the manufacturers’ instructions. Poly(A) RNA-Seq libraries were constructed using the NEB Ultra II RNA Library Prep kit. DNA and RNA sequencing was performed by the Scientific Operations core at the WSI on Pacific Biosciences SEQUEL II (HiFi), Illumina HiSeq 4000 (RNA-Seq) and Illumina NovaSeq 6000 (10X) instruments. Hi-C data were also generated from muscle tissue of xgPatDepr1 using the Arima2 kit and sequenced on the Illumina NovaSeq 6000 instrument.

### Genome assembly, curation and evaluation

Assembly was carried out with Hifiasm ( Cheng *et al.*, 2021) and haplotypic duplication was identified and removed with purge_dups ( Guan *et al.*, 2020). One round of polishing was performed by aligning 10X Genomics read data to the assembly with Long Ranger ALIGN, calling variants with FreeBayes ( Garrison & Marth, 2012). The assembly was then scaffolded with Hi-C data ( Rao *et al.*, 2014) using SALSA3 ( Ghurye *et al.*, 2019). The assembly was checked for contamination and corrected using the gEVAL system ( Chow *et al.*, 2016) as described previously ( Howe *et al.*, 2021). Manual curation was performed using gEVAL, HiGlass ( Kerpedjiev *et al.*, 2018) and Pretext ( Harry, 2022). The mitochondrial genome was assembled using MitoHiFi ( Uliano-Silva *et al.*, 2023), which runs MitoFinder ( Allio *et al.*, 2020) or MITOS ( Bernt *et al.*, 2013) and uses these annotations to select the final mitochondrial contig and to ensure the general quality of the sequence.

A Hi-C map for the final assembly was produced using bwa-mem2 ( Vasimuddin *et al.*, 2019) in the Cooler file format ( Abdennur & Mirny, 2020). To assess the assembly metrics, the *k*-mer completeness and QV consensus quality values were calculated in Merqury ( Rhie *et al.*, 2020). This work was done using Nextflow ( Di Tommaso *et al.*, 2017) DSL2 pipelines “sanger-tol/readmapping” ( Surana *et al.*, 2023a) and “sanger-tol/genomenote” ( Surana *et al.*, 2023b). The genome was analysed within the BlobToolKit environment ( Challis *et al.*, 2020) and BUSCO scores ( Manni *et al.*, 2021; Simão *et al.*, 2015) were calculated.

Table 3 contains a list of relevant software tool versions and sources.

**Table 3.** Software tools: versions and sources.

Software tool	Version	Source
BlobToolKit	4.2.1	https://github.com/blobtoolkit/blobtoolkit
BUSCO	5.3.2	https://gitlab.com/ezlab/busco
FreeBayes	1.3.1	https://github.com/freebayes/freebayes
gEVAL	N/A	https://geval.org.uk/
Hifiasm	0.15	https://github.com/chhylp123/hifiasm
HiGlass	1.11.6	https://github.com/higlass/higlass
Long Ranger ALIGN	2.2.2	https://support.10xgenomics.com/genome-exome/software/pipelines/latest/advanced/other-pipelines
Merqury	MerquryFK	https://github.com/thegenemyers/MERQURY.FK
MitoHiFi	2	https://github.com/marcelauliano/MitoHiFi
PretextView	0.2	https://github.com/wtsi-hpag/PretextView
purge_dups	1.2.5	https://github.com/dfguan/purge_dups
SALSA	3	https://github.com/salsa-rs/salsa
sanger-tol/genomenote	v1.0	https://github.com/sanger-tol/genomenote
sanger-tol/readmapping	1.1.0	https://github.com/sanger-tol/readmapping/tree/1.1.0

### Genome annotation

The Ensembl gene annotation system ( Aken *et al.*, 2016) was used to generate annotation for the *Patella depressa* assembly (GCA_948474765.1). Annotation was created primarily through alignment of transcriptomic data to the genome, with gap filling via protein-to-genome alignments of a select set of proteins from UniProt ( UniProt Consortium, 2019).

### Wellcome Sanger Institute – Legal and Governance

The materials that have contributed to this genome note have been supplied by a Darwin Tree of Life Partner. The submission of materials by a Darwin Tree of Life Partner is subject to the **‘Darwin Tree of Life Project Sampling Code of Practice’**, which can be found in full on the Darwin Tree of Life website here. By agreeing with and signing up to the Sampling Code of Practice, the Darwin Tree of Life Partner agrees they will meet the legal and ethical requirements and standards set out within this document in respect of all samples acquired for, and supplied to, the Darwin Tree of Life Project.

Further, the Wellcome Sanger Institute employs a process whereby due diligence is carried out proportionate to the nature of the materials themselves, and the circumstances under which they have been/are to be collected and provided for use. The purpose of this is to address and mitigate any potential legal and/or ethical implications of receipt and use of the materials as part of the research project, and to ensure that in doing so we align with best practice wherever possible. The overarching areas of consideration are:

•   Ethical review of provenance and sourcing of the material

•   Legality of collection, transfer and use (national and international) 

Each transfer of samples is further undertaken according to a Research Collaboration Agreement or Material Transfer Agreement entered into by the Darwin Tree of Life Partner, Genome Research Limited (operating as the Wellcome Sanger Institute), and in some circumstances other Darwin Tree of Life collaborators.

## Data Availability

European Nucleotide Archive: *Patella depressa* (black-footed limpet). Accession number PRJEB45666; https://identifiers.org/ena.embl/PRJEB45666 ( Wellcome Sanger Institute, 2023). The genome sequence is released openly for reuse. The *Patella depressa* genome sequencing initiative is part of the Darwin Tree of Life (DToL) project. All raw sequence data and the assembly have been deposited in INSDC databases. Raw data and assembly accession identifiers are reported in Table 1.

## Author information

Members of the Marine Biological Association Genome Acquisition Lab are listed here: https://doi.org/10.5281/zenodo.8382513.

Members of the Darwin Tree of Life Barcoding collective are listed here: https://doi.org/10.5281/zenodo.4893703.

Members of the Wellcome Sanger Institute Tree of Life Management, Samples and Laboratory team are listed here: https://doi.org/10.5281/zenodo.10066175.

Members of Wellcome Sanger Institute Scientific Operations: Sequencing Operations are listed here: https://doi.org/10.5281/zenodo.10043364.

Members of the Wellcome Sanger Institute Tree of Life Core Informatics team are listed here: https://doi.org/10.5281/zenodo.10066637.

Members of the Tree of Life Core Informatics collective are listed here: https://doi.org/10.5281/zenodo.5013541.

Members of the Darwin Tree of Life Consortium are listed here: https://doi.org/10.5281/zenodo.4783558.
