# Supplementary material for: The genome sequence of the black-footed limpet, Patella depressa (Pennant, 1777)
Source: Wellcome Open Res. 2024 Feb 19;9:47. [Version 1] doi: 10.12688/wellcomeopenres.20687.1 (PMC11109591; doi:10.12688/wellcomeopenres.20687.1)
Supplement: Peer review history

# Reviewer report: Reviewer response for version 1

**DOI:** 10.21956/wellcomeopenres.22892.r80209

**Published:** 2024-05-15

**Authors:** Tim Regan

**Affiliations:** 1 University of Edinburgh, Edinburgh, UK

This is a clear and concise genome report for an important rocky shore species. The data availability along with the methods used are well presented.

 Context for the ecological importance of this species along with its natural range were nicely presented in the Background introduction. I just noticed a few sentences in this section which could be rewritten to avoid ambiguity:

 "It is absent from Ireland as it did not cross the Irish Sea at the end of the last Ice Age"

 I presume this is conjecture rather than established fact?

 "Patella depressa is capable of being a multiple brooder (Ribeiro et al., 2009). This pattern contrasts to only developing one brood in the cooler 1940s (Orton & Southward, 1961). "

 in the 1940s, which were cooler? 

 "P. depressa has a shorter larval life (Ribiero, 2009) than P. vulgata, and can be reared without feeding, suggesting at least partial lecitrophy."

 Were these anoxic growing conditions? Could another possibility could be that the larvae feed on microbes during these early stages?

Are sufficient details of methods and materials provided to allow replication by others?

Yes

Is the rationale for creating the dataset(s) clearly described?

Yes

Are the datasets clearly presented in a useable and accessible format?

Yes

Are the protocols appropriate and is the work technically sound?

Yes

Reviewer Expertise:

Molluscan genomics, immunology, aquaculture.

I confirm that I have read this submission and believe that I have an appropriate level of expertise to confirm that it is of an acceptable scientific standard.

# Reviewer report: Reviewer response for version 1

**DOI:** 10.21956/wellcomeopenres.22892.r80203

**Published:** 2024-05-13

**Authors:** Rafael Zardoya

**Affiliations:** 1 Departamento de Biodiversidad y Biología Evolutiva, Museo Nacional de Ciencias Naturales (MNCN-CSIC), Madrid, Spain

This report presents the nine-chromosome genome of the black-footed limpet. The different statistics show that the genome is of high quality in terms of accuracy and contiguity. The procedures to sequence, assemble and scaffold the new genome, as explained in the material & methods section, are standard and adequate. Altogether, this is a new reference genome very useful for studies on comparative genomics and evolution of gastropods. As suggestion, in the first lines of the background section, after mentioning the confused taxonomy of the species, I would include a reference to the molecular phylogeny of the genus:

 Sá-Pinto et al. (2005) [Ref-1]

 It would be also useful comparing the results with the numbers (e.g., size, N50, etc.) associated to other recently published genomes of Patella:

 Halstead-Nussloch et al. (2024) [Ref-2]

 Hawkins SJ et al. ( 2023) [Ref-3]

 Lawniczak M et al. (2022) [Ref-4]

Are sufficient details of methods and materials provided to allow replication by others?

Yes

Is the rationale for creating the dataset(s) clearly described?

Yes

Are the datasets clearly presented in a useable and accessible format?

Yes

Are the protocols appropriate and is the work technically sound?

Yes

Reviewer Expertise:

phylogenomics, comparative genomics, transcriptomics, gastropods

I confirm that I have read this submission and believe that I have an appropriate level of expertise to confirm that it is of an acceptable scientific standard.

## References

[1] : Phylogeny and phylogeography of the genus Patella based on mitochondrial DNA sequence data. *Journal of Experimental Marine Biology and Ecology* .2005;325(1) : 10.1016/j.jembe.2005.04.025 95-110 10.1016/j.jembe.2005.04.025

[2] : The genome of the rayed Mediterranean limpet Patella caerulea (Linnaeus, 1758). *Genome Biol Evol* .2024;16(4) : 10.1093/gbe/evae070 10.1093/gbe/evae070 38546725 PMC11003540

[3] : The genome sequence of the common limpet, Patella vulgata (Linnaeus, 1758). *Wellcome Open Res* .2023;8: 10.12688/wellcomeopenres.20008.1 418 10.12688/wellcomeopenres.20008.1 37994322 PMC10663661

[4] : The genome sequence of the blue-rayed limpet, Patella pellucida Linnaeus, 1758. *Wellcome Open Res* .2022;7: 10.12688/wellcomeopenres.17825.1 126 10.12688/wellcomeopenres.17825.1 36874573 PMC9975397

# Reviewer report: Reviewer response for version 1

**DOI:** 10.21956/wellcomeopenres.22892.r77912

**Published:** 2024-04-12

**Authors:** David E.K. Ferrier

**Affiliations:** 1 School of Biology, University of St Andrews, St Andrews, Scotland, UK

The rationale is clear. This limpet species is a significant member of rocky-shore coastal ecosystems and is a useful indicator species for environmental changes such as warming.

 The various protocols are appropriate and clearly described, with helpful links provided to supplement the descriptions provided. The work is certainly technically sound.

 The materials and methods descriptions meet accepted standards for these data notes, and the datasets such as the assemblies of the two haplotypes are accessible and useable.

 I spotted just a single typo. In Background, second paragraph: I think “lecitrophy” should be changed to “lecithotrophy”.

Are sufficient details of methods and materials provided to allow replication by others?

Yes

Is the rationale for creating the dataset(s) clearly described?

Yes

Are the datasets clearly presented in a useable and accessible format?

Yes

Are the protocols appropriate and is the work technically sound?

Yes

Reviewer Expertise:

NA

I confirm that I have read this submission and believe that I have an appropriate level of expertise to confirm that it is of an acceptable scientific standard.
